# Evaluation of a Temperature/Humidity Data Logger for the Usage in Cattle Barns †

**DOI:** 10.3390/s24227117

**Published:** 2024-11-05

**Authors:** Malina Flessner, Felix König, Christian Guse, Michael Iwersen, Daniela Klein-Jöbstl

**Affiliations:** Veterinary Systems Transformation and Sustainability, Clinical Department Farm Animals and Food System Science, University of Veterinary Medicine Vienna, Veterinaerplatz 1, 1210 Vienna, Austria; malina.flessner@vetmeduni.ac.at (M.F.); felix.koenig@vetmeduni.ac.at (F.K.); christian.guse@vetmeduni.ac.at (C.G.); michael.iwersen@vetmeduni.ac.at (M.I.)

**Keywords:** temperature/humidity data logger, THI, heat stress, climate change, precision livestock farming, animal welfare

## Abstract

Climate change is a worldwide problem that is manifested in livestock farming with a decrease in animal health and welfare and economic losses due to heat stress. Therefore, a precise and continuous recording of the barn climate is essential to be able to implement actions at a certain threshold. The aim of this study was to evaluate a logger for temperature and humidity (Kestrel Drop D2) marketed for on-farm use in comparison to various other temperature/humidity data loggers under field conditions. Four different sensors were used and placed in different settings in cattle barns to correlate temperature and humidity measurements. Data were recorded for over a year in total. The data were very highly correlated. Furthermore, the area under the curve for the evaluated logger in comparison to the other ones was 0.99 to 1.0, using a temperature–humidity index cut-off of 72, often set to define heat stress. In conclusion, the evaluated logger performed equally well as the other used devices. For on-farm use, it is suitable.

## 1. Introduction

Climate change has had influences on the weather worldwide and continues to impact the earth increasingly. Heat stress periods will occur more often and last longer [1]. For cattle, heat stress leads to a decreased milk yield, lower reproduction rates, an increased risk of metabolic diseases, and has a negative impact on the well-being of the animals [2,3,4,5]. The actual extent of global warming is difficult to forecast, since it depends on various factors that reinforce each other. Thus, the climate prognoses for the next decades include several possible scenarios [6]. Accordingly, risk simulations for heat stress in cattle are important for the future of dairy farming and have been carried out [7].

The most used method to define thresholds for heat stress in animals is the temperature–humidity index (THI), which was first introduced as an index for heat-related discomfort for people by Thom in 1959 [8]. Subsequently, it was adopted to determine heat stress in cattle [9]. Different formulas exist to calculate the THI [10,11]. Schüller et al. (2014) [12] suggest using the formula by Kendall and Webster (2009) [11], which is applicable to all climatic zones. For farmers, it is essential to know when the THI exceeds a threshold that is out of the cows’ thermal comfort zone [13] so that they can offer shade, ventilation, and spray evaporation as possible cooling mechanisms to alleviate heat stress [14,15]. It has been shown that the meteorological data from weather stations on nearby farms differ widely from the climate conditions inside of the stables [16]. Consequently, for farmers and for studies on heat stress in cattle, it is important to measure the temperature and relative humidity inside of the barn where the animals are housed.

Available climate loggers for on-farm use either directly provide a calculated THI or the THI has to be calculated by the user from the raw data. When comparing the THI of different devices and studies, the underlying formula must be taken into account.

A THI logger for everyday use in barns should be reliable, cheap, easy to install, offer real-time data directly on a device, and ideally come with an alarm function. There are different temperature and humidity loggers on the market that are used by farmers and in studies [4,17,18,19,20,21,22]. Generally, there is a lack in commercially available sensor systems with independent validation in farm environments [23,24]. This is also true for temperature and relative humidity loggers. This leads to the need for more extensive validation of sensor systems in farming environments [24].

So far, we do not have a gold standard for temperature and relative humidity measurement in the field. Consequently, in studies, different sensors are compared for evaluation [25].

One THI logger fulfilling the aforementioned criteria for use in cattle barns that has already been used in several studies is the Kestrel Drop D2 (KE, Nielsen-Kellerman Co., Boothwyn, PA, USA) [17,21]. To date, however, no independent validation of the device has been reported.

The aim of this study was to evaluate if the Kestrel Drop D2 logger marketed for on-farm use in cattle barns provides reliable environmental data for farmers and research.

## 2. Materials and Methods

In this evaluation, the KE was compared with several other temperature and humidity data loggers. The utilized loggers and systems are outlined in Table 1.

The KE is a compact, portable, battery-operated device measuring temperature with a resolution of 0.1 °C and an accuracy of ±0.5 °C, as well as relative humidity with a resolution of 0.1% and an accuracy of ±2%, according to the manufacturer. The data can be accessed wirelessly with a dedicated application on mobile devices using standard Bluetooth connection. The GPS location needs to be enabled to view or download the data, but no internet connection is required. The KE shows the temperature, relative humidity, THI, dew point, and heat index in real-time.

Another portable device used in this study is the Tinytag Plus 2 TGP-4500 (TT, Gemini Data Loggers (UK) Ltd., Chichester, UK), which is a battery-powered temperature and humidity logger. For setup and data collection, a specific USB cable and computer software (Tinytag Explorer 6.0) are necessary.

The Papago TH3 Temperature and Humidity Sensor (PP, Papouch s.r.o., Prague, Czech Republic) must be connected to a communication device (Papago Meteo ETH, in this study, available in different versions), which is powered over ethernet (PoE) or with an external power adapter. Data transfer can be set up individually through access using either RS-482, ethernet, or the IEEE (2020) standard [26].

The HOBO RXW-THC-B-868 (HOBO, Onset Computer Corp., Bourne, MA, USA) was the fourth sensor used in this study, and it is a battery-operated sensor that requires a connection to a data logger and sends data wirelessly using a frequency of 868 MHz. The data are stored in cloud solution from the manufacturer and can be accessed either using Web interface or API.

**Table 1:** Data sheets of the various loggers used in this study according to the manufacturers.

Sensor Name	Kestrel Drop D2 AG Livestock Heat Stress Monitor (KE)	Tinytag Plus 2 TGP-4500 (TT)	Papago Meteo ETH Papago TH3 Temperature and Humidity Sensor (PP)	HOBO RXW-THC-B-868 HOBOnet Wireless Temp/RH Sensor (HOBO)
Company name	Nielsen-Kellerman Co. (Boothwyn, PA, USA)	Gemini Data Loggers (UK) Ltd. (Chichester, UK)	Papouch s.r.o. (Prague, Czechia)	Onset Computer Corp. (Bourne, MA, USA)
Temperature range	−10 to 55 °C	−25 to 85 °C	−40 to 125 °C	−40 to 75 °C
Temperature accuracy	±0.5 °C	±0.4 to 0.9 °C ^1^	±0.3 to 0.5 °C	±0.25 to 0.20 °C
Temperature resolution	0.1 °C	0.01 °C	0.1 °C	0.02 °C
RH ^2^ range	10 to 90%	0 to 100%	0 to 100%	0 to 100%
RH accuracy	±2%	±3%	±2%	±2.5 to 3.5%
RH resolution	0.1%	0.3%	0.1%	0.01%
Measuring interval	2 s to 12 h	1 s to 10 d	up to 5 per s	1 min to 18 h
Data storage	7275 readings (10 min: 50 d)	32,000 readings (10 min: 222 d)	online (backup on device: 120 entries)	cloud (backup on device: 16 MB)
Data transfer	Bluetooth, app	USB cable, software (Tinytag Explorer 6.0)	cable, continuous	868 MHz
Power source	CR2032 battery	LS 14250 battery	PoE ^3^ or external power	2 AA 1.5 V lithium batteries
Battery life (if applicable)	3 to 4 months	1 year		1 year
Sealing	waterproof	waterproof	-	weatherproof
Shock resistance	yes	-	-	-
Size	24 × 46 × 60 mm	34 × 57 × 80 mm	40 × 16 × 10 mm, cable (various lengths) and logger	46 × 11 × 10 mm
Weight	34 g	110 g	5 m cable: 192 g, logger: 130 g	sensor: 223 g, logger: 2.2 kg
Validation	against NIST [27,28] ^4^ standards	according to ISO [29]	sensors tested with transfer standards by Sensirion	tested and found with the limits of a class B digital device

^1^ Accuracy depends on the temperature. ^2^ RH = relative humidity, ^3^ PoE = power over ethernet, ^4^ NIST = National Institute of Standards and Technology.

The loggers were used and compared in four different settings. Table 2 shows the set-up, loggers used, and the amount and interval of data collection for each setting.

The first setting was a “controlled setting.” The loggers (KE1, KE2, PP, and TT), were placed next to each other in a closed styrofoam box, with no direct sunlight or air movement exposure. For testing the sensor in various cattle barns, three on-farm settings with different devices were selected. In the 2nd setting, the loggers were installed in a closed cattle barn on a pillar near the feed bunk, opposite the animal area (out of animal reach). The PP and TT were immediately next to each other. The KE was at the same height, but approximately 20 cm away from the other two loggers. In this setting, the loggers were not exposed to direct sunlight, but air movement along the feed bunk (all loggers in the same “wind” direction). Setting 3 was in an open climate calf barn, in a group box housing up to 15 calves between the ages of two and twelve weeks. The loggers were positioned immediately next to each other under a wooden shed with plastic curtains. The loggers were positioned at the height of the animals’ heads, protected with a wire mesh fence. The loggers were not exposed to direct sunlight or wind. In the 4th setting, the loggers were installed in an open dairy cattle barn, positioned on a pillar in the area of the feed bunk, opposite the animal area (out of animal reach). Both loggers were fixed directly under each other. In this setting, the loggers were not exposed to direct sunlight, but air movement along the feed bunk. Pictures showing the settings can be found in the Supplementary Materials (Figure S1). None of the loggers were directly exposed to sunlight, heating, or cooling systems.

All loggers (KE, TT, PP, and HOBO) were time synchronized either with the atomic clock or over GPS. The KE, TT, and HOBO log once per minute, or every 10 min. The PP logs repeatedly per minute, and the data were summarized and averaged according to the logging frequency of the KE.

All statistical analyses were performed with either Excel (version 2402, Microsoft 365, Microsoft Corp., Redmond, WA, USA), SPSS (version 28, IBM Corp., Armonk, NY, USA), or R (version 4.4.0, R Core Team, Vienna, Austria), with the R packages ggplot2 (3.5.1), Cairo (1.6.2), dplyr (1.1.4), and tidyr (1.3.1). Data were excluded from the evaluation if the temperature and humidity measurements of the KE were missing.

Data on temperature and relative humidity measured with the devices were used. For all devices, the THI was calculated using the formula of Kendall and Webster, 2009 [12]. All data were tested for normality using the Kolmogorov–Smirnoff test. Considering the data were not normally distributed, non-parametric tests were applied. In Setting 1, two KEs were used to validate the reproducibility (agreement) of the loggers. Data recorded with the different devices were compared using the Spearman Rho correlation coefficient [30]. Precision was defined by the Spearman Rho correlation and R^2^. This was calculated for all settings between all devices. Furthermore, the area under the curve-receiver operating characteristics (AUC-ROC) analysis for THI, with a cut-off at ≥72 for heat stress [31], was calculated for KE with either TT and PP (settings 1–3) or HOBO (Setting 4) as a comparison method.

## 3. Results

**Figure 1:**
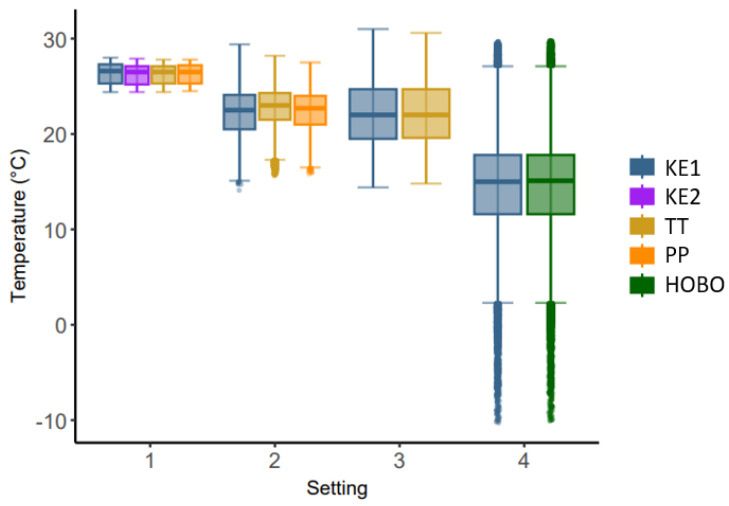
Temperature in °C measured with the Kestrel Drop D2 AG in different settings. Setting 1: controlled setting in a styrofoam box in an office, 2: closed dairy cattle barn, 3: calf barn, 4: open dairy cattle barn.

**Figure 2:**
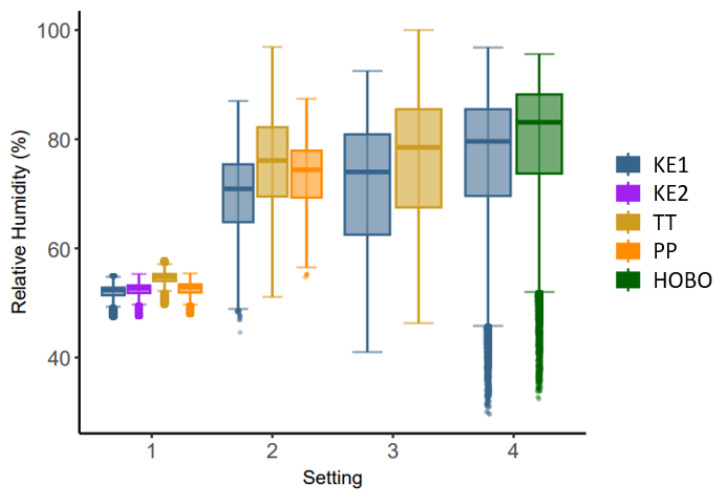
Relative humidity in % measured with the Kestrel Drop D2 AG in different settings. Setting 1: controlled setting in a styrofoam box in an office, 2: closed dairy cattle barn, 3: calf barn, 4: open dairy cattle barn.

Overall, 266,115 temperature and humidity measurements were collected. Due to data losses in KE, overall, 33,219 temperature and relative humidity datapoints of the other loggers had to be excluded. The temperature and relative humidity ranges differed between settings (Figure 1 and Figure 2). The descriptive statistics can be found in the Supplementary Materials (Table S1).

The correlation coefficients of the temperature between the different sensors are presented in Table 3. All of the utilized loggers highly correlated with the KE, varying between 0.999 (HOBO) and 0.975 (PP).

Table 4 displays the correlation of the relative humidity obtained with the devices used.

Table 5 illustrates the Spearman Rho correlation coefficient for temperature, humidity, and THI, according to the different settings, compared with the KE.

**Figure 3:**
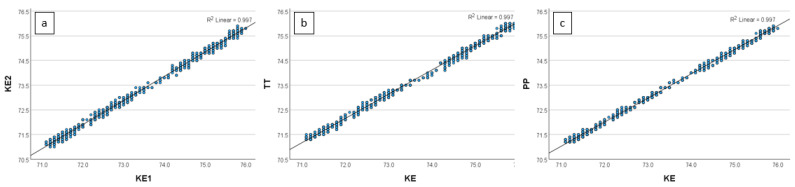
(**a**–**c**) Scatterplot of the relationship of the temperature–humidity index (THI) between (**a**) two different KEs (Kestrel Drop D2 AG Livestock Heat Stress Monitor; **KE1** and KE2), (**b**) **KE1** and the TT (Tinytag Plus 2 TGP-4500), and (**c**) **KE1** and the PP (Papago Meteo ETH and Papago TH3 Temperature and Humidity Sensor) in Setting 1 (controlled setting in a closed styrofoam box in an office.

**Figure 4:**
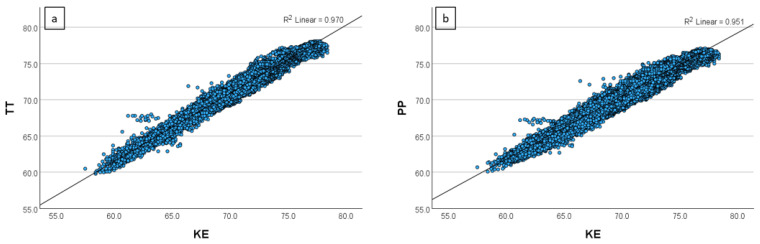
(**a**,**b**) Scatterplot of the relationship of the temperature–humidity index (THI) between (**a**) the KE and the TT (Tinytag Plus 2 TGP-4500) and (**b**) the KE and the PP (Papago Meteo ETH and Papago TH3 Temperature and Humidity Sensor) in Setting 2 (closed dairy cattle barn).

**Figure 5:**
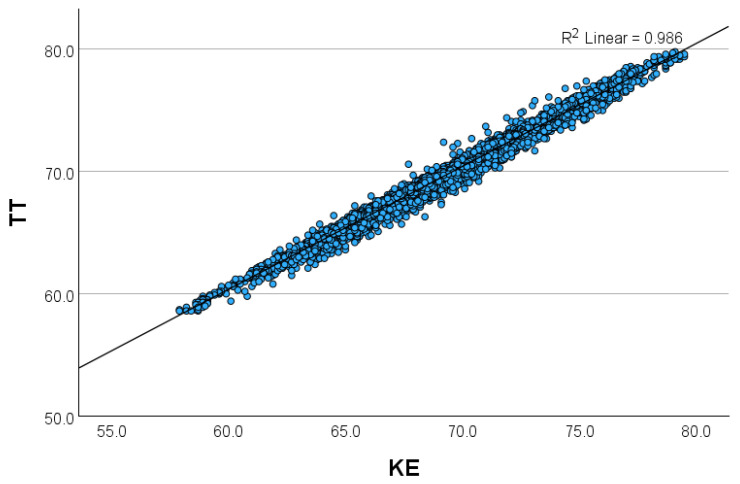
Scatterplot of the relationship of the temperature–humidity index (THI) between the KE (Kestrel Drop D2 AG Livestock Heat Stress Monitor) and the PP (Papago Meteo ETH and Papago TH3 Temperature and Humidity Sensor) in Setting 3 (open calf barn).

**Figure 6:**
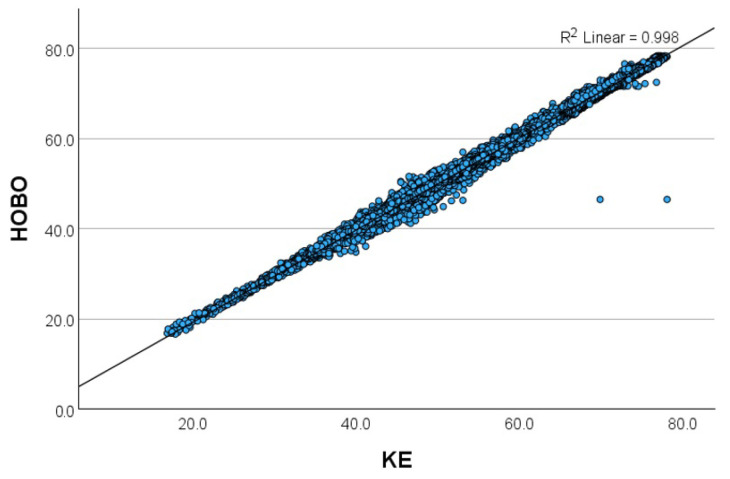
Scatterplot of the relationship of the temperature–humidity index (THI) between the KE (Kestrel Drop D2 AG Livestock Heat Stress Monitor) and the HOBO (HOBO RXW-THC-B-868 HOBOnet Wireless Temp/RH Sensor) in Setting 4 (open dairy cattle barn).

Figure 3, Figure 4, Figure 5 and Figure 6 show the relationship between the THIs (temperature–humidity indexes) [12] of the KE and the THIs of the other loggers.

**Figure 7:**
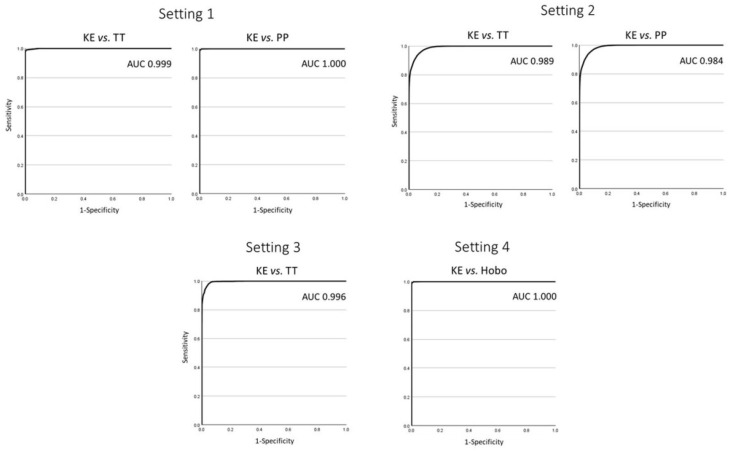
AUC-ROC with a THI cut-off of ≥72.0 for the KE (Kestrel Drop D2 AG Livestock Heat Stress Monitor) in comparison to the TT (Tinytag Plus 2 TGP-4500), PP (Papago Meteo ETH and Papago TH3 Temperature and Humidity Sensor), or HOBO (HOBO RXW-THC-B-868 HOBOnet Wireless Temp/RH Sensor) in the four different settings.

The results of the AUC-ROC for the four different settings are presented in Figure 7.

## 4. Discussion

With the increasing importance of climatic stress, especially heat stress, in cattle, it is important to measure the barn climate (temperature and relative humidity) and calculate the THI using a reliable method.

For this purpose, we compared different loggers and systems that have already been used in the field and in scientific studies in cattle barns [4,17,18,19,20,21,22]. The Kestrel Drop D2 is marketed for on-farm use. To the best of our knowledge, this logger has not been evaluated scientifically so far. In comparison to other on-farm methods, this logger has the advantage of being relatively cheap, the data can be accessed and visualized via Bluetooth on a portable device in real-time, and also has THI and alarms. Furthermore, the data are saved and can be exported easily. The disadvantages are that, compared to the other methods, less data can be stored, and the batteries must be changed on a regular basis. During this study, we also experienced data loss during the data transfer process. The reasons for the data loss during the download could be interference with other devices that also operate on BLE frequency. Additionally, the battery status indicator was not accurate. In some KEs, the batteries discharged rather quickly after we changed them.

Overall, the agreement was very high. In a controlled setting (Setting 1, in a polyfoam box in an office), the methods showed perfect agreement [32] for temperature and relative humidity, as well as the calculated THI. In on-farm settings, slight differences between the loggers and the settings were observed. Possible explanations for these differences could be dirt, soil, and moisture on the logger (see Figure S2 in the Supplementary Materials). Nonetheless, we compared the data recorded using a clean, newly installed logger with the data revealed after some time, and, when the device was dirty, and could not find any significant differences. Another possible reason could be that, although the loggers were positioned in direct proximity, differences in moisture, air speed, etc., may have existed. Overall, our evaluations revealed very good to perfect agreement between the different loggers, in the different settings, and at different logging intervals.

The THI revealed very good agreement between the devices. For farmers to be able to act upon heat stress, a threshold for alarms is vital. In cattle, a THI of 72 is a value often used to define the onset of heat stress [4,33]. We used this limit for the AUC-ROC analysis, and the KE distinguishes almost perfectly between heat stress and no heat stress compared with the other used devices (TT, PP, and HOBO).

In conclusion, the evaluated KE performed equally well as the other methods used. For on-farm use, the KE is suitable, because the farmer can see the climate data live on their mobile device when in the Bluetooth range of the logger. The option to receive alerts for a certain THI is convenient. Additionally, the logger is cheap and sturdy, so multiple loggers could be installed in various locations on the farm. On the other hand, the KE is less suitable for study purposes, due to a short battery life, a low storage capacity, and the risk of data loss.

Given the good results of temperature and relative humidity measurements, the KE can be recommended for scientific studies. However, due to its relatively short battery life, limited storage capacity, and the potential for data loss, it is advisable to use alternative loggers for research. If using the KE, it is essential to closely monitor the loggers to minimize the risk of data losses.

## Figures and Tables

**Table 2:** The four different settings for the evaluation.

Setting	1	2	3	4
Location	Controlled setting Closed styrofoam box in an office	Closed dairy cattle barn in Austria	Open calf barn in Austria	Open dairy cattle barn in northern Germany
KE ^1^ compared to logger	KE, PP ^2^, TT ^3^	TT, PP	TT	HOBO ^4^
Logging frequency of KE	Every minute	Every minute, every 10 min	Every 10 min	Every minute, every 10 min
Time period	August 2021	June to September 2021	July to August 2021	July 2021 to May 2022
Data points	6118	48,809	4022	173,947

^1^ Kestrel Drop D2 AG Livestock Heat Stress Monitor, ^2^ Tinytag Plus 2 TGP-4500, ^3^ Papago Meteo ETH and Papago TH3 Temperature and Humidity Sensor, ^4^ HOBO RXW-THC-B-868 HOBOnet Wireless Temp/RH Sensor. All of the loggers in the barns were protected from animals and rain. Battery changes were necessary, according to the measuring interval set, and varied between the three battery-powered loggers.

**Table 3:** Correlation of the temperature between the KE (Kestrel Drop D2 AG) and the other used sensors.

	KE1 ^1^ Compared to	KE2	TT ^2^	PP ^3^	HOBO ^4^
Setting 1	r ^5^	0.997	0.996	0.998	-
	R^2 6^	0.997	0.996	0.998	-
	ME ^7^	0.124	0.127	0.07	-
	RMSE ^8^	0.140	0.148	0.092	-
	MAE ^9^	0.3	0.4	0.2	-
	N ^10^	6118	6118	6118	-
Setting 2	r	-	0.987	0.968	-
	R^2^	-	0.978	0.944	-
	ME	-	0.398	0.583	-
	RMSE	-	0.493	0.687	-
	MAE	-	4.3	4.1	-
	N	-	41,062	48,781	-
Setting 3	r	-	0.991	-	-
	R^2^	-	0.984	-	-
	ME	-	0.344	-	-
	RMSE	-	0.430	-	-
	MAE	-	1.9	-	-
	N	-	4022	-	-
Setting 4	r	-	-	-	0.999
	R^2^	-	-	-	0.998
	ME	-	-	-	0.181
	RMSE	-	-	-	0.263
	MAE	-	-	-	21.2
	N	-	-	-	173,947

^1^ Kestrel Drop D2 AG Livestock Heat Stress Monitor, ^2^ Tinytag Plus 2 TGP-4500, ^3^ Papago Meteo ETH and Papago TH3 Temperature and Humidity Sensor, ^4^ HOBO RXW-THC-B-868 HOBOnet Wireless Temp/RH Sensor, ^5^ Correlation coefficient of Spearman Rho, ^6^ Coefficient of determination, ^7^ Mean absolute error, ^8^ Root mean squared error, ^9^ Maximum absolute error, ^10^ Sample size.

**Table 4:** Correlation of the relative humidity between the different sensors and the KE (Kestrel Drop D2 AG).

	KE1 ^1^ Compared to	KE2	TT ^2^	PP ^3^	HOBO ^4^
Setting 1	r ^5^	0.988	0.987	0.990	-
	R^2 6^	0.995	0.991	0.995	-
	ME ^7^	0.288	2.483	0.485	-
	RMSE ^8^	0.321	2.491	0.169	-
	MAE ^9^	0.8	3.4	0.8	-
	N ^10^	6118	6118	6118	-
Setting 2	r	-	0.938	0.903	-
	R^2^	-	0.884	0.828	-
	ME	-	6.162	3.805	-
	RMSE	-	6.831	4.585	-
	MAE	-	26.4	18.6	-
	N	-	41,062	48,781	-
Setting 3	r	-	0.975	-	-
	R^2^	-	0.960	-	-
	ME	-	5.039	-	-
	RMSE	-	5.531	-	-
	MAE	-	19	-	-
	N	-	4022	-	-
Setting 4	r	-	-	-	0.945
	R^2^	-	-	-	0.940
	ME	-	-	-	3.969
	RMSE	-	-	-	4.591
	MAE	-	-	-	38.2
	N	-	-	-	173,947

^1^ Kestrel Drop D2 AG Livestock Heat Stress Monitor, ^2^ Tinytag Plus 2 TGP-4500, ^3^ Papago Meteo ETH and Papago TH3 Temperature and Humidity Sensor, ^4^ HOBO RXW-THC-B-868 HOBOnet Wireless Temp/RH Sensor, ^5^ Correlation coefficient of Spearman Rho, ^6^ Coefficient of determination, ^7^ Mean absolute error, ^8^ Root mean squared error, ^9^ Maximum absolute error, ^10^ Sample size.

**Table 5:** Spearman Rho correlation coefficient for the temperature, the relative humidity, and the THI (temperature–humidity index) between the loggers and the KE (Kestrel Drop D2 AG) in the four different settings.

Category	Loggers	Setting			
		1	2	3	4
Temperature	KE2 ^1^	0.997	-	-	-
	TT ^2^	0.996	0.987	0.991	-
	PP ^3^	0.998	0.968	-	-
	HOBO ^4^	-	-	-	0.999
Relative Humidity	KE2	0.988	-	-	-
	TT	0.987	0.938	0.975	-
	PP	0.990	0.903	-	-
	HOBO	-	-	-	0.945
Temperature–Humidity Index	KE2	0.997	-	-	-
	TT	0.997	0.983	0.993	-
	PP	0.998	0.974	-	-
	HOBO	-	-	-	0.999

^1^ Kestrel Drop D2 AG Livestock Heat Stress Monitor, ^2^ Tinytag Plus 2 TGP-4500, ^3^ Papago Meteo ETH and Papago TH3 Temperature and Humidity Sensor, ^4^ HOBO RXW-THC-B-868 HOBOnet Wireless Temp/RH Sensor.

## Data Availability

The raw data supporting the conclusions of this article will be made available by the authors upon request.

## Supplementary Materials

The following supporting information can be downloaded at: https://www.mdpi.com/article/10.3390/s24227117/s1, Figure S1: Position of the logger in the different settings. Figure S2: Sensor covered in dirt. Table S1: Descriptive statistics for the different loggers in the four settings.
