# Supplementary material for: Evaluation of a Temperature/Humidity Data Logger for the Usage in Cattle Barns
Source: Sensors (Basel). 2024 Nov 5;24(22):7117. doi: 10.3390/s24227117 (PMC11598373; doi:10.3390/s24227117)
Supplement: Supplementary file 1 [file sensors-24-07117-s001.zip › Supplementary Materials_Fig S1.pdf]

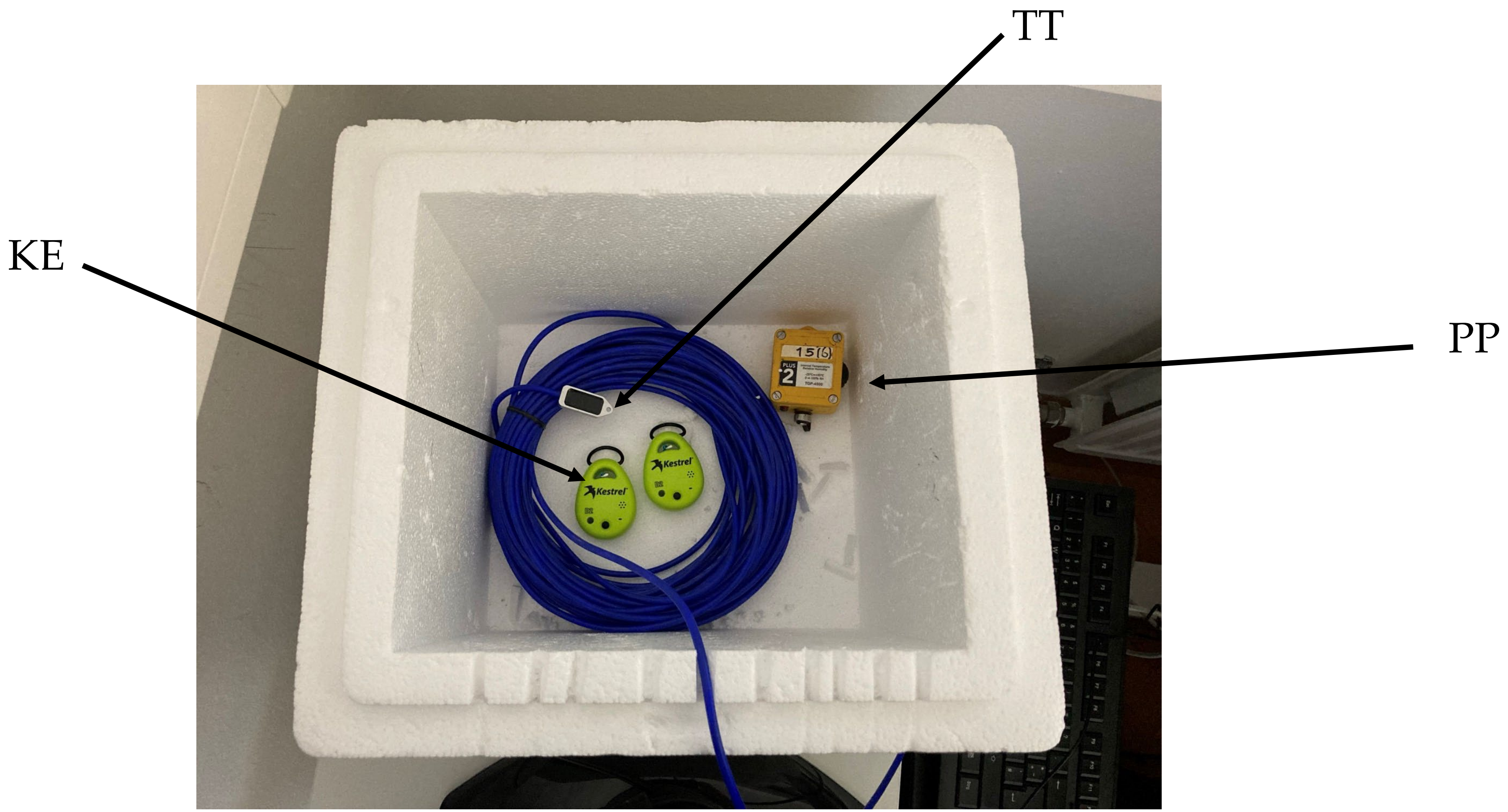

Figure S1 <u>Setting 1</u> Controlled setting. Closed styrofoam box in an office with no direct sun or air movement exposure. Two Kestrel Drop D2 AG Livestock Heat Stress Monitor (KE) were compared to a Tinytag Plus 2 TGP-4500 (TT) and a Papago Meteo ETH Papago TH3 Temperature and Humidity Sensor (PP).

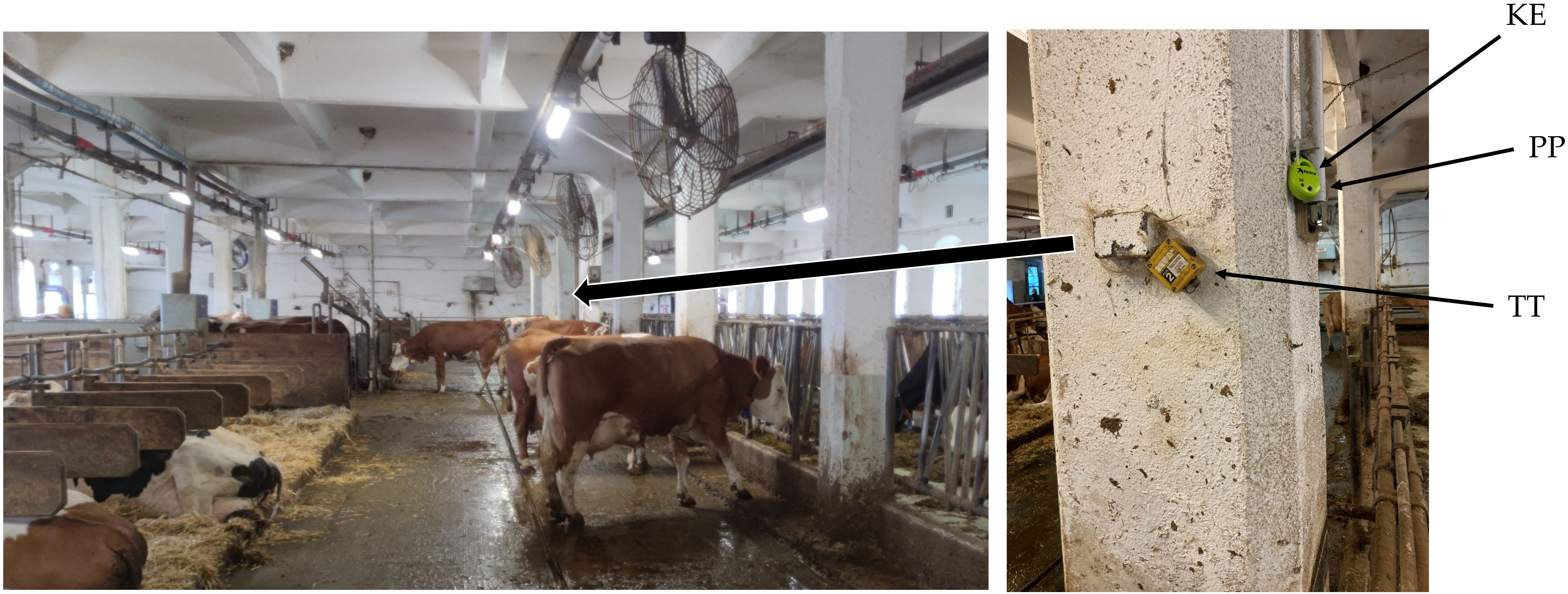

Figure S1 <u>**Setting 2**</u> in a closed cattle barn at a pillar in the area of the feed bunk, opposite to the animal area (out of animal reach). In this setting the loggers were not exposed to direct sun, but air movement along the feed bunk. The Kestrel Drop D2 AG Livestock Heat Stress Monitor (KE) was compared to a Tinytag Plus 2 TGP-4500 (TT) and a Papago Meteo ETH Papago TH3 Temperature and Humidity Sensor (PP).

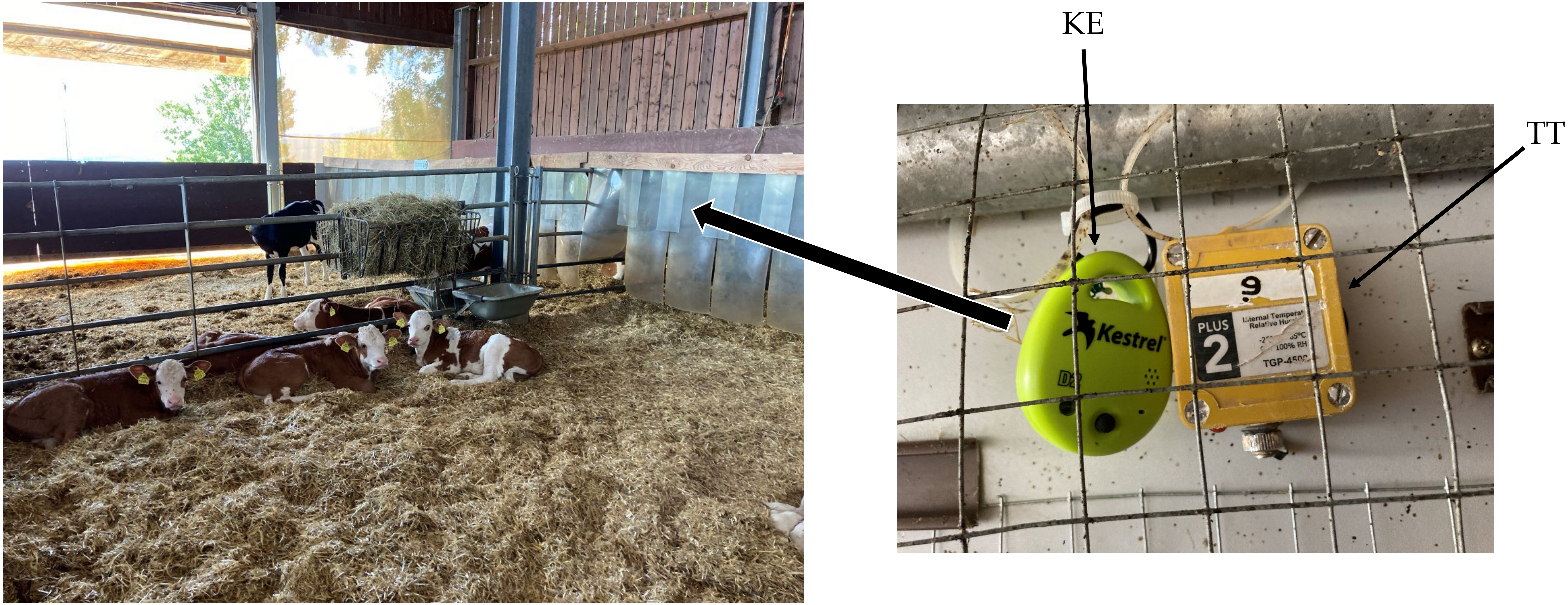

Figure S1 **Setting 3 i**n an open climate calf barn, in a group box housing up to 15 pre-weaned calves (2 to 12 weeks old). The loggers were positioned under a wooden shed with plastic curtains*, at the height of the animals head, protected by a wire mesh fence from the calves. In this setting the loggers were not exposed to direct sun or wind. The Kestrel Drop D2 AG Livestock Heat Stress Monitor (KE) was compared to a Tinytag Plus 2 TGP-4500 (TT).

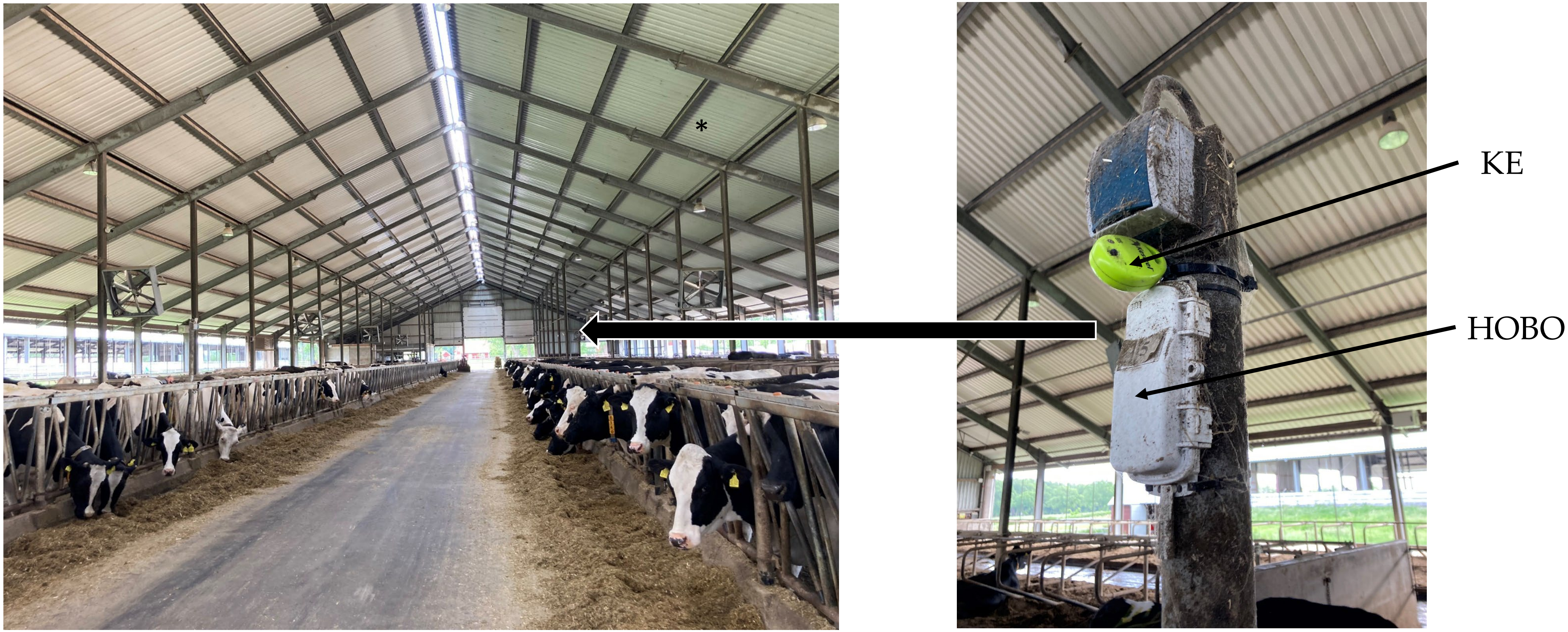

Figure S1 **Setting 4** in an open dairy cattle barn, positioned at a pillar in the area of the feed bunk, opposite to the animal area (out of animal reach). In this setting the loggers were not exposed to direct sun, but air movement along the feed bunk. The Kestrel Drop D2 AG Livestock Heat Stress Monitor (KE) was compared to a HOBO RXW-THC-B-868 HOBOnet Wireless Temp/ RH Sensor (HOBO).
