# Supplementary figures and images for: Evaluation of a Temperature/Humidity Data Logger for the Usage in Cattle Barns
Source: Sensors (Basel). 2024 Nov 5;24(22):7117. doi: 10.3390/s24227117 (PMC11598373; doi:10.3390/s24227117)

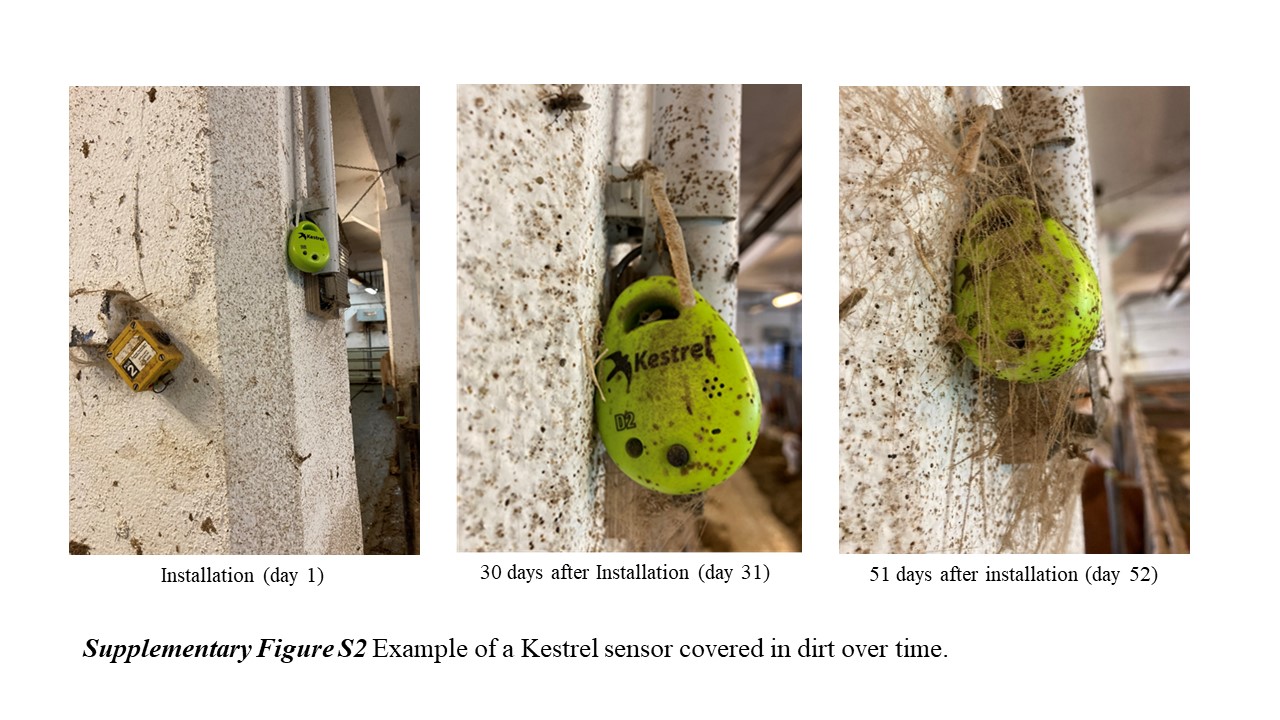

Supplement: Supplementary file 1 [file sensors-24-07117-s001.zip › Supplementary Materials_Fig S2.jpg]
